# On Syrupus Iodidi Ferri

**Published:** 1851-01

**Authors:** 


*On Syrupus Iodidi Ferri.*—Mr. Tizier, Apothecary, of Dublin, makes the following useful suggestions, on the preparation of this valuable article:

The new Dublin pharmacopœia directs "to introduce the Iodine, iron and water, into a glass flask, and apply a *moderate heat* until the solution loses its red color." Now, as the great success of the first part of the process depends on the rapidity with which it is conducted, without any unnecessary exposure to the air (but what cannot be avoided) until a neutral solution be affected, there is evidently considerable time lost, and danger of decomposition incurred by pursuing these directions. This circumstance appears more remarkable, when we are aware that all extraneous application of heat is superfluous. Iodine and iron exert so powerful an affinity for each other in the presence of water, as to combine with the greatest facility, generating a large amount of sensible heat; at the same time it is only necessary then to bring their particles *constantly in immediate contact with each other*, to fulfil this end to our entire satisfaction, and for this purpose, two practical points must be attended to, first, to *rotate* the flask containing the mixed substances briskly and diligently for some moments in the hand, until the deep red color of the solution disappears, and is succeeded by its olive-green pellucid and normal one, which, when tested, should be perfectly permanent to the action of amidine. And, secondly, to break down the iron turnings as small as possible, carefully freed from any adhering oxide, by which means a greater superficial extent is exposed to chemical action, and thus ensure rapidity of combination more easily.

By careful attention to these apparently insignificant points, the preparation, in its first stage, will be divested of much of its pracical difficulty, and rendered easier of execution, while risk from decomposition will be entirely obviated. Care should be taken to filter the solution

(after testing it) into the saccharine mass, which preserves the neutral iodide of iron so far as to preclude the possibility of change from any subsequent heat that may be employed.—*Pharmaceutical Journal, November* 1 1850.

